# Current research on carbetocin and implications for prevention of postpartum haemorrhage

**DOI:** 10.1186/s12978-018-0529-0

**Published:** 2018-06-22

**Authors:** Fiona J. Theunissen, Lester Chinery, Yeshita V. Pujar

**Affiliations:** 1 Concept Foundation, Geneva, Switzerland; 2 Department of Obstetrics & Gynaecology, KLE Academy of Higher Education and Research’s J. N. Medical College, Belagavi, Karnataka India

**Keywords:** Carbetocin, Clinical trials, Heat stable, Prevention, Postpartum haemorrhage, PPH, Oxytocin, Uterotonics

## Abstract

**Background:**

Postpartum haemorrhage (PPH) is the leading cause of maternal mortality in low-income countries and is a significant contributor to severe maternal morbidity and long-term disability. Carbetocin may be an underused uterotonic for prevention of PPH. A number of studies are being conducted that may challenge the place of oxytocin as the first choice of uterotonics for prevention of PPH. This paper describes the current research into carbetocin and ranking of effectiveness of uterotonics that may provide important new information to assist healthcare decision makers to ensure that women receive an effective uterotonic for prevention of PPH.

**Methods:**

We searched the WHO International Clinical Trials Registry Platform for current studies on effectiveness of carbetocin for prevention of PPH following vaginal delivery with sample sizes large enough to provide quality evidence to support potential changes to international guidelines. We also searched the Cochrane Library for current systematic reviews including carbetocin used in prevention of PPH.

**Results:**

Susceptibility to degradation from exposure to heat is one of the key causes of reduced effectiveness of oxytocin in preventing PPH from uterine atony. Although heat stable and effective in preventing PPH, misoprostol is also subject to degradation due to exposure to moisture and produces some side-effects. Other uterotonics (including ergometrine and combinations of oxytocin, ergometrine and misoprostol) are also available and used with varying safety and effectiveness profiles and quality issues. Efforts to reduce maternal mortality from PPH include research studies seeking to identify safe, stable, effective uterotonics. Heat stable carbetocin is the subject of two major clinical studies into its effectiveness in preventing PPH following vaginal deliveries, information that could expand its application for prevention of PPH.

**Conclusion:**

Heat stable carbetocin is being investigated as a potential alternative to oxytocin. This paper describes two current clinical trials on carbetocin and a network meta-analysis ranking of all uterotonic agents, including carbetocin, which combined may provide evidence supporting expansion of the use of the heat stable formulation of carbetocin in PPH prevention.

## Background

Postpartum haemorrhage (PPH) is the leading cause of maternal mortality in low-income countries [1] and is a significant contributor to severe maternal morbidity and long-term disability, as well as to a number of other severe maternal conditions, generally associated with more substantial blood loss, including severe anaemia, cardiac failure and sepsis.

Currently, the World Health Organization (WHO) [2] recommends Active Management of the Third Stage of Labour (AMTSL) to prevent PPH. AMTSL as a prophylactic intervention is composed of a package of three components or steps: 1) administration of a uterotonic, preferably oxytocin, immediately after birth of the baby; 2) controlled cord traction (CCT) to deliver the placenta; and 3) massage of the uterine fundus after the placenta is delivered [2]. The administration of a uterotonic to the mother immediately after the birth of the baby is identified as the most important step [2]. Oxytocin is the recommended uterotonic where its efficacy can be assured.

The effectiveness of uterotonics in generating the uterine contractions necessary to prevent haemorrhage can be impaired through exposure to conditions that cause the uterotonic to degrade. In many low- and middle-income countries where access to sustained cold-chain is unavailable, the efficacy of oxytocin cannot be assured because it is susceptible to heat degradation [3, 4]. Other uterotonics include ergometrine/methylergometrine, misoprostol and fixed-dose combinations of these uterotonics. Ergometrine degrades when exposed to heat or light [3]. Misoprostol degrades rapidly when exposed to moisture [5]. When degraded, the level of active ingredient is decreased, resulting in reduced effectiveness.

Carbetocin has been widely used for prevention of PPH since 1997 [6]. Innovation in the manufacture of carbetocin has resulted in a product which meets the International Council for Harmonisation of Technical Requirements for Pharmaceuticals for Human Use (ICH) stability requirements for hot and humid climates (Zone IV A and B) for 36 months at 30 °C and 6 months at 40 °C [6]. In countries where cold chain is unreliable or not available, a safe and stable uterotonic could contribute to reduction of maternal mortality from PPH. Despite this, carbetocin is not one of the uterotonics recommended for prevention of PPH by the WHO. This is due primarily to the lack of evidence on its effectiveness in prevention of PPH following vaginal deliveries.

### Objective

The global health community has long sought an effective uterotonic for prevention of PPH that withstands degradation. The objective of this research paper is to highlight studies into heat stable carbetocin, a molecule which may be sufficiently clinically effective and structurally stable to be included in the list of recommended uterotonics for prevention of PPH.

### Search method

We searched the WHO International Clinical Trials Registry Platform for current studies on effectiveness of carbetocin for prevention of PPH following vaginal delivery with sample sizes large enough to provide quality evidence to support potential changes to international guidelines. We also searched the Cochrane Library for current systematic reviews including carbetocin used in prevention of PPH.

## Results

In 2018, results of two major clinical trials on the effectiveness of carbetocin in prevention of PPH following vaginal deliveries will be published:

- *Heat stable carbetocin (formerly carbetocin RTS) for preventing postpartum haemorrhage: a randomized non-inferiority, controlled trial – the World Health Organization (CTRI/2016/05/006969 - Protocol). Sample size 30,000 women* [7]
- *Intramuscular Oxytocics: A Comparison Study of Intramuscular Carbetocin, Syntocinon and Syntometrine for the Third Stage of Labour Following Vaginal Birth (IMox) – North Bristol NHS Trust (NCT02216383 - Protocol). Sample size 6285 women* [8]

We also identified a systematic review ranking all uterotonics on the basis of effectiveness and side-effects:

- *Uterotonic agents for preventing postpartum haemorrhage: a network meta-analysis – Cochrane Review* [9]

These three studies will provide the evidence needed to include (heat stable) carbetocin in global recommendations and respond to the need for additional options for the prevention of PPH.

## Main text

Globally, access to effective uterotonics remains a key barrier to reducing maternal mortality. In 2012, the UN Commission on Life Saving Medicines [10] reported on the quality of 13 reproductive and maternal health medicines, highlighting serious quality problems in uterotonics across low and middle-income countries. In 2016, the *Quality of oxytocin available in low- and middle-income countries: a systematic review of the literature*, published in BJOG found that across the literature reviewed, on average 45.6% of oxytocin samples failed quality tests mostly due to insufficient amounts of active pharmacological ingredient [11].

A further study adding to the evidence on poor quality of uterotonics in low- and middle-income countries was published by the United States Pharmacopoeia (USP) Promoting the Quality of Medicines Program in BMC Pregnancy and Birth in January 2018 [12]. In this study, titled *Quality medicines in maternal health: results of oxytocin, misoprostol, magnesium sulfate and calcium gluconate quality audits,* the researchers found that 74.2% of oxytocin injection samples and 33.7% of misoprostol samples failed the assay test [12].

Additional activities focussed on highlighting the need for action on improving the quality and effectiveness of uterotonics for prevention of postpartum haemorrhage include the October 2017 Technical Consultation on Messaging for Management of Oxytocin organised by the USAID Global Health Supply Chain Program in collaboration with the Reproductive Health Supplies Coalition. The consultation involved technical experts from the WHO, UNFPA and a number of other organizations. The meeting report, *A Current Review of Evidence* will be available online in March 2018 and will be followed by an in-depth review of the evidence on oxytocin quality. An outcome of the technical consultation was the release of an advocacy messaging framework aimed at improving the quality of oxytocin at the point of use called “Buy Quality Oxytocin, Keep it Cold” [13].

While effort is going into improving the quality of oxytocin including advocacy, manufacturing quality improvements, regulatory strengthening and supply chain and storage improvements, research is being conducted into whether (heat stable) carbetocin is an underutilized option for prevention of PPH.

Following are summaries of the current major research activities on effectiveness of carbetocin for prevention of PPH and how they could influence guidelines and practices.

### Heat stable carbetocin for preventing postpartum haemorrhage: a randomized non-inferiority, controlled trial (CTRI/2016/05/006969 - protocol) [7]

The research objective of this trial is to evaluate if heat stable carbetocin (formerly carbetocin RTS) 100 μg intramuscular (IM) is non-inferior to oxytocin 10 IU IM, in preventing PPH in women delivering vaginally. Uterine atony is the principle cause of PPH. Administration of an effective uterotonic after delivery of the baby has been demonstrated to reduce PPH caused by uterine atony. The majority of deaths due to PPH could be avoided through the use of prophylactic uterotonics during the third stage of labour [7].

Oxytocin (IM/IV, 10 IU) is recommended as the uterotonic drug of choice. The WHO and other organizations recommend that oxytocin should be stored between 2-8C° to prevent degradation and loss of effectiveness [14]. Carbetocin, widely used for the prevention of PPH following caesarean section, is a more stable molecule [6] and induces a prolonged uterine response, when administered postpartum [15]. A manufacturer of carbetocin has developed a stable formulation (heat stable carbetocin, previously referred to as carbetocin RTS) which makes it a potential option for countries where maintaining the cold chain is problematic [6, 16].

Twenty-three centres from 10 countries (India, Nigeria, Kenya, Uganda, United Kingdom, Egypt, South Africa, Singapore, Argentina and Thailand) participated in the trial. A total of 29,658 participants were enrolled globally. The blinding of the trial required that both products were refrigerated, although the heat stable carbetocin compound was used.

This trial was conducted by the WHO, who completed enrolment at the end of January 2018 and who are currently analysing data and preparing the results. Should this trial demonstrate that heat stable carbetocin is non-inferior to oxytocin in preventing PPH, the WHO will have evidence to support including heat stable carbetocin in its Recommendations for the Prevention and Treatment of PPH and in the WHO Model List of Essential Medicines.

The trial is part of a broader collaboration involving regulatory, advocacy and manufacturing activities between Merck for Mothers (MSD for Mothers outside the USA and Canada), Ferring Pharmaceuticals and the World Health Organization (WHO) to make heat stable carbetocin accessible, pending trial results, in the public sector of low and lower-middle income countries at an affordable and sustainable price.

### Intramuscular Oxytocics: a comparison study of intramuscular Carbetocin, Syntocinon and Syntometrine for the third stage of labour following vaginal birth (IMox) (NCT02216383 - protocol) [8]

The Intramuscular Oxytocics trial aims to compare the effectiveness, side-effects and cost of three uterotonics – carbetocin, oxytocin (Syntocinon) and the fixed dose combination of oxytocin/ergometrine (Syntometrine). All products were refrigerated for this trial to preserve blinding. The trial is important because of the additional comparison with the oxytocin/ergometrine combination, widely used because of its effectiveness in preventing PPH, but which is associated with a number of side-effects.

Studies in the use of carbetocin following caesarean section have demonstrated effectiveness in prevention of PPH and a safety and side-effects profile similar to oxytocin [17]. No studies have directly compared all three medicines or compared their overall cost. The investigators planned to recruit 6285 women, in four maternity units in the United Kingdom, with recruitment ending in 2018.

The study’s primary outcome is the proportion of patients requiring additional uterotonic drugs after administration of study drug. Investigators will perform an analysis of cost effectiveness once all results are available. The results of this trial will provide additional support for any changes to international guidelines.

### Uterotonic agents for preventing postpartum haemorrhage: a network meta-analysis [9]

The objective of the *Uterotonic agents for preventing postpartum haemorrhage: a network meta-analysis* is to assess the clinical effectiveness and side-effect profile of uterotonic drugs to prevent PPH and to generate a clinically useful ranking of available uterotonics according to their effectiveness and side-effects [9]. A scientifically rigorous ranking could reduce uncertainty about which is the most effective drug for preventing PPH.

All uterotonic drugs used for prevention of PPH have been compared with each other including oxytocin, misoprostol, ergometrine, carbetocin, oxytocin plus misoprostol, oxytocin plus ergometrine and placebo or no-treatment.

The study included all randomised controlled individual or cluster trials evaluating effectiveness or side-effects of uterotonic drugs for preventing PPH identified from the Cochrane’s Pregnancy and Childbirth Group (PCG) trials register. The interventions considered were uterotonics administered by healthcare providers during the third stage of labour for preventing PPH compared with a control uterotonic or with placebo or no treatment. The targeted population was women having a vaginal birth or a caesarean section in hospitals or community settings.

The study found that, based on published studies, ergometrine plus oxytocin combination, carbetocin, and misoprostol plus oxytocin combination were most effective in preventing PPH ≥ 500 mL. Ergometrine plus oxytocin combination was most effective in preventing PPH ≥ 1000 mL. Carbetocin had the most favourable side-effect profile amongst the top three options. The carbetocin evidence came from small studies. The authors plan to update the study when the two major carbetocin study results are published. [9]

The network meta-analysis (and the forthcoming update) are of particular value in providing information on the relative effectiveness and side-effects of uterotonics whether they have been directly compared in a clinical trial or not, and will therefore assist decision makers and clinicians in determining the best option for particular patient populations.

## Conclusion

Heat Stable Carbetocin is being investigated as a potential alternative to oxytocin and is the subject of two large clinical trials for use in prevention of PPH in vaginal deliveries. In this discussion, we provided an overview of the two clinical trials on (heat stable) carbetocin and the meta-analysis ranking of all uterotonic agents, including carbetocin.

The WHO trial (Heat stable carbetocin for prevention of PPH) is the largest trial ever conducted on uterotonics for prevention of PPH and is clearly designed to support guideline changes. The IMox trial (A Comparison Study of Intramuscular Carbetocin, Syntocinon and Syntometrine for the Third Stage of Labour Following Vaginal Birth), though smaller is still one of the larger clinical trials in this field and the results will be important.

The outcomes of these three pieces of research are likely to result in a change in the WHO Recommendations for the Prevention and Treatment of PPH to include carbetocin as an option for prevention of PPH. The drug may also be included in the WHO Model List of Essential Medicines. Governments and clinicians may have an option to assist in the prevention of maternal mortality resulting from preventable PPH that is heat stable and therefore more effective at the time of use in setting where cold chain is not reliable.

## Abbreviations

AMTSL: Active management of the third stage of labour
BMC: BioMed Central
IM: Intramuscular
IMox: Intramuscular oxytocics
IV: Intravenous
MSD: Merck sharp & dohme corp., a subsidiary of merck & Co
NHS: National health service
PPH: Postpartum hemorrhage
RTS: Room temperature stable
UNFPA: United Nations population fund
USAID: United States agency for international development
WHO: World Health Organization
